# Identification of TRIM21 and TRIM14 as Antiviral Factors Against Langat and Zika Viruses

**DOI:** 10.3390/v17050644

**Published:** 2025-04-29

**Authors:** Pham-Tue-Hung Tran, Mir Himayet Kabir, Naveed Asghar, Matthew R. Hathaway, Assim Hayderi, Roger Karlsson, Anders Karlsson, Travis Taylor, Wessam Melik, Magnus Johansson

**Affiliations:** 1 School of Medical Science, Faculty of Medicine and Health, Örebro University, SE-70362 Örebro, Sweden; hungtpt2210@gmail.com (P.-T.-H.T.); naveed.asghar@oru.se (N.A.); assim.hayderi@oru.se (A.H.); wessam.melik@oru.se (W.M.); 2 Department of Medical Microbiology and Immunology, College of Medicine and Life Sciences, University of Toledo, Toledo, OH 43612, USA; mirhimayet.kabir@rockets.utoledo.edu (M.H.K.); matthew.hathaway@rockets.utoledo.edu (M.R.H.); travis.taylor@utoledo.edu (T.T.); 3 Department of Infectious Diseases, Institute of Biomedicine, Sahlgrenska Academy of the University of Gothenburg, SE-40234 Gothenburg, Sweden; roger.karlsson@nanoxisconsulting.com; 4 Department of Clinical Microbiology, Sahlgrenska University Hospital, SE-41346 Gothenburg, Sweden; 5 Nanoxis Consulting AB, SE-40016 Gothenburg, Sweden; anders.karlsson@nanoxisconsulting.com

**Keywords:** flavivirus, antiviral host factor, TRIM14, TRIM21, TRIM38, ZIKV, LGTV, WNV, NS3, NS5

## Abstract

Flaviviruses are usually transmitted to humans via mosquito or tick bites, whose infections may lead to severe diseases and fatality. During intracellular infection, they remodel the endoplasmic reticulum (ER) membrane to generate compartments scaffolding the replication complex (RC) where replication of the viral genome takes place. In this study, we purified the ER membrane fraction of virus infected cells to identify the proteins that were enriched during flavivirus infection. We found that tripartite motif-containing proteins (TRIMs) including TRIM38, TRIM21, and TRIM14 were significantly enriched during infection with mosquito-borne (West Nile virus strain Kunjin and Zika virus (ZIKV)) and tick-borne (Langat virus (LGTV)) flaviviruses. Further characterizations showed that TRIM21 and TRIM14 act as restriction factors against ZIKV and LGTV, while TRIM38 hinders ZIKV infection. These TRIMs worked as interferon-stimulated genes to mediate IFN-I response against LGTV and ZIKV infections. Restriction of ZIKV by TRIM14 and TRIM38 coincides with their colocalization with ZIKV NS3. TRIM14-mediated LGTV restriction coincides with its colocalization with LGTV NS3 and NS5 proteins. However, TRIM21 did not colocalize with ZIKV and LGTV NS3 or NS5 protein suggesting its antiviral activity is not dependent on direct targeting the viral enzyme. Finally, we demonstrated that overexpression of TRIM21 and TRIM14 restricted LGTV replication.

## 1. Introduction

The *Orthoflavivirus* genus consists of 53 recognized species [1], many of which are known to cause severe disease in humans and contribute to an annual infection risk to over half of the global population [2]. Within the genus, (re)emerging viruses such as dengue virus (DENV), Zika virus (ZIKV), West Nile virus (WNV), Japanese encephalitis virus (JEV), yellow fever virus (YFV), and members of the tick-borne encephalitis virus (TBEV) serogroup, including Langat virus (LGTV), pose significant threats to global health. These viruses can cause severe disease manifestations, ranging from hemorrhagic fever to encephalitis, leading to substantial morbidity and mortality and placing a heavy burden on healthcare systems worldwide. These viruses are enveloped and contain a positive-sense, single-stranded RNA genome that encodes a single polyprotein. Cleavages of the polyprotein by host and viral proteases result in individual viral proteins, including the structural capsid (C), pre-membrane (prM), and envelope (E) proteins, and seven nonstructural (NS) proteins (NS1, NS2A, NS2B, NS3, NS4A, NS4B, and NS5) important for the formation of the replication complex (RC) [3,4,5,6], viral replication [7,8], and assembly [9,10,11] at the endoplasmic reticulum (ER). Two of the NS proteins have enzymatic activity: NS5 possesses both methyltransferase (MTase) and RNA-dependent RNA polymerase (RdRp) activities important for viral replication, whereas the NS3 protein encodes the viral RNA helicase and, together with its co-factor NS2B (NS2B/3), functions as the viral protease required for the cleavage of the flavivirus polyprotein [12].

Flavivirus’ ability to avoid or evade host antiviral responses is essential to establish their replication and transmission [13]. However, it is not fully understood how different flaviviruses avoid host innate immunity. Host-specific interactions with the interferon (IFN) response have been demonstrated for flaviviruses that can only antagonize IFN-dependent signaling in the context of primate hosts [14]. However, the IFN-stimulated genes (ISGs) that contribute to the host-specific restriction of flaviviruses are not well characterized. Upon sensing viral RNA, cells produce type I IFN, which functions as a crucial hinderance against virus infection. IFN signaling upregulates the expression of ISGs to establish an antiviral state. ISGs can directly target viral products for degradation or act on pathways employed by viruses to limit the infection [14,15,16,17,18,19]. Among ISGs, many tripartite motif-containing proteins (TRIMs) are powerful, direct-acting antiviral restriction factors or modulators of the cellular response to infection [20]. More than 100 TRIMs exist in the human genome, many of which are found as antiviral restriction factors for flaviviruses [21]. We have previously demonstrated that TRIM5α and TRIM79α restricted tick-borne flavivirus replication by inducing the proteasomal degradation of NS3 and NS5, respectively [22,23].

In the current study, we demonstrated three TRIM proteins (TRIM14, TRIM21, and TRIM38) as antiviral restriction factors for flaviviruses. TRIM14 has been demonstrated to possess a strong capability of regulating IFN and NF-kB induction in the host defense against influenza virus A (IAV), Ebola virus (EBOV), and hepatitis C virus (HCV) [24,25,26]. It has been shown that TRIM21 plays an important role in regulating the immune response during viral infections [27,28,29]. Another important TRIM family member, TRIM38, has been shown to enhance the antiviral effect of IFN treatment against hepatitis B virus (HBV) by enhancing the expression of antiviral proteins [30].

Virus replication within the RC is recognized by host pattern recognition receptors to trigger IFN-mediated antiviral responses [14,15]. Despite previous studies on the role of TRIM family proteins in viral restriction, their specific functions during flavivirus infection remain incompletely understood. In the current study, we systematically screened for proteins that were induced and recruited to the RC after infection with the WNV strain Kunjin (WNV_KUN_), ZIKV, and LGTV. The WNV_KUN_ and ZIKV were used as the mosquito-borne flavivirus (MBFV) models, while the LGTV was used as the tick-borne flavivirus (TBFV) model. We identified that TRIM14, TRIM21, and TRIM38 were significantly enriched during WNV_KUN_, ZIKV, and LGTV infection.

TRIM14, TRIM21, and TRIM38 restricted ZIKV, whereas only TRIM14 and TRIM21 inhibited LGTV replication. The TRIM14- and TRIM38-mediated restriction of ZIKV coincided with their colocalization with the viral protease NS3, while the TRIM14-mediated LGTV restriction was linked to interactions with both NS3 and NS5. In contrast, TRIM21 did not colocalize with viral proteins, suggesting that its antiviral activity is independent of the direct targeting of viral enzymes. By identifying these TRIM proteins as antiviral restriction factors, our study provides new insights into the host regulation of flavivirus infections. These findings enhance our understanding of the molecular determinants influencing human susceptibility to flavivirus infection and may inform future therapeutic strategies.

## 2. Materials and Methods

### 2.1. Cell Culture

Vero (ATCC), HEK-293T, and A549 (ATCC) cells were maintained in Dulbecco’s Modified Eagle’s Medium (DMEM) containing 1 g/L glucose (Gibco, Paisley, UK) supplemented with 5% heat-inactivated fetal bovine serum (HI–FBS) (Gibco) and 100 U/mL penicillin–streptomycin (PEST) (Gibco) at 37 °C in 5% CO_2_.

### 2.2. Viruses

The LGTV strain TP21, ZIKV, and WNV_KUN_ were used in this study. Flavivirus working stocks were propagated, and infectious titers were calculated by plaque assay on Vero cells. The multiplicity of infection (MOI) for flavivirus infections is represented as plaque forming unit (PFU) per cell. All procedures with LGTV, ZIKV, and WNV_KUN_ were performed in biosafety level 2 laboratories.

### 2.3. ER Purification and Enrichment

A549 cells were infected by WNV_KUN_, ZIKV, or LGTV with 0.1 MOI. After 72 h, infected cells were UV-irradiated for 20 min to inactivate the virus, before ER purification, as previous described [31]. In brief, cells were harvested through trypsinization and homogenized using Dounce tissue grinders. The total lysates were then centrifuged at 15,000× *g* 4 °C for 10 min. The resulting supernatants were fractionated by ultracentrifugation after being loaded onto a discontinuous sucrose gradient (2 M, 1.5 M, and 1.3 M) and centrifuged at 152,000× *g* 4 °C for 70 min, which yielded a distinct ER membrane band at the interface of the 1.3 M sucrose layer. The ER membrane fraction was then collected and subjected to ultracentrifugation at 126,000× *g* at 4 °C for 45 min. The ER membrane pellets were air-dried and finally resuspended in PBS. To enrich proteins interacting with the flavivirus replication complex, 300 μg of the purified ER fraction was incubated with 4 μg of NS1 antibody for ZIKV (Abcam, Cambridge, UK) and LGTV (Mybiosouce, San Diego, CA, USA) and NS4A antibody for WNV_KUN_ (GeneTex, Hsinchu City, Taiwan). Subsequently, 50 μL of MACSflex™ MicroBeads (MACS, Stockholm, Sweden) was added, and the mixture was incubated at 4 °C for 1 h. The mixture was loaded on μ columns (MACS) holding a μMACS separator to retain the microbeads on the columns. The beads were washed four times with PBS at 4 °C. Finally, bead-binding proteins were eluted in PBS by removing the columns from the μMACS separator.

### 2.4. Tryptic Digestion of Proteins and Tandem Mass Tag (TMT) Labeling for Quantitative Proteomics

Samples were digested using trypsin 5 µg/mL in PBS (Gibco) containing 0.5% SDC (sodium deoxycholate from a 5% stock solution in de-ionized water). Trypsin was added from a stock solution of 20 µg/mL prepared according to the manufacturer’s instructions. The samples were digested for 15 h. SDC originating from the tryptic digest was removed by acidification with 10% TFA and centrifugation. The pH of the peptides in the supernatant was adjusted with 1M TEAB for the subsequent labeling using tandem mass tag (TMT) reagents (Thermo Scientific, Rockford, IL, USA) according to the manufacturer’s instructions. The labeled samples were combined and concentrated using vacuum centrifugation. The combined TMT-labeled sample was fractionated into 20 primary fractions by basic reversed-phase chromatography (bRP-LC) using a Dionex Ultimate 3000 UPLC system (Thermo Fisher Scientific, Waltham, MA, USA). Peptide separations were performed using a reversed-phase XBridge BEH C18 column (3.5 μm, 3.0 × 150 mm, Waters Corporation, Milford, MA, USA) and a linear gradient from 3% to 40% solvent B over 18 min followed by an increase to 100% B over 5 min. Solvent A was 10 mM ammonium formate buffer at pH 10.00, and solvent B was 90% acetonitrile, 10% 10 mM ammonium formate at pH 10.00. The primary fractions were concatenated into 10 fractions, evaporated and reconstituted in 15 μL of 3% acetonitrile, 0.2% formic acid for nLC MS analysis.

### 2.5. Liquid Chromatography with Tandem Mass Spectrometry (LC-MS/MS) Analysis

The fractions were analyzed on an QExactive HF mass spectrometer interfaced with the Easy-nLC1200 liquid chromatography system (Thermo Fisher Scientific). Peptides were trapped on an Acclaim Pepmap 100 C18 trap column (100 μm × 2 cm, particle size: 5 μm, Thermo Fischer Scientific) and separated on an in-house packed analytical column (75 μm × 30 cm, particle size: 3 μm, Reprosil-Pur C18, Dr. Maisch, Entringen, Ammerbuch, Germany) using a gradient from 5% to 80% acetonitrile in 0.2% formic acid over 90 min at a flow of 300 nL/min. The instrument operated in data-dependent mode, where the precursor ion mass spectra were acquired at a resolution of 60,000, *m*/*z* range: 380–1200. The 10 most intense ions with charge states 2 to 5 were selected for fragmentation using HCD at stepped collision energy settings of 35. The isolation window was set to 0.8 Da and the dynamic exclusion to 20 s and 10 ppm. MS2 spectra were recorded at a resolution of 60,000 with the maximum injection time set to 110 ms.

### 2.6. Proteomic Data Analysis

Proteomic data analysis was performed using Proteome Discoverer version 2.4 (Thermo Scientific). The database matching was performed using the Mascot search engine v. 2.5.1 (Matrix Science) against a custom database containing SwissProt *Homo sapiens* and proteins from WNV_KUN_, ZIKV, or LGTV. The precursor mass tolerance was set to 5 ppm and the fragment ion tolerance to 0.2 Da. Trypsin was selected as the enzyme with 0 missed cleavages, cysteine methylthiolation was set as a fixed modification, and methionine oxidation and TMT11plex on lysine and peptide N-termini were set as a variable modification. Percolator was used for the PSM validation with the strict FDR threshold of 1%. For quantification, TMT reporter ions were identified in the MS2 HCD spectra. Only the unique identified peptides were considered for the relative quantification. No normalization was used in the search, and reporter ion abundances for the samples were used for the evaluation of the results. An additional processing node, importing protein annotations of biological processes, cellular components, and molecular functions, was enabled. Only proteins significantly detected (FDR < 0.01) in all three replicates of each virus infection were used in the data analysis. GO enrichment analysis was performed using ToppFun with the detected proteins (FDR < 0.01 and fold change > 1.5) from the ZIKV-infected ER samples compared to the noninfected ER control (https://toppgene.cchmc.org, accessed on 31 January 2020).

### 2.7. Gene Constructs, Transfections, and Virus Infection

Mammalian expression plasmids pcDNA3.1+/C-DYK for TRIM14 with the (K)DYK tag (Genscript, Leiden, The Netherlands), pcDNA3.1+/C-DYK for TRIM21 with the (K)DYK tag (Genscript), and pcDNA3.1+/C-DYK for TRIM38 with the Myc-DDK-tag gene construct (Origene, Rockville, MD, USA) were used in this study. These constructs contain a neomycin gene conferring G418 antibiotic resistance for cell selection. To establish cell lines overexpressing these TRIMs, the DNA constructs were linearized, followed by transfection into A549 cells by using a Nucleofector system (Lonza, Basel, Switzerland). Two days after transfection, the cell culture was supplemented with 600 µg/mL G418 (Invivogen, Toulouse, France) to select transfected cells expressing TRIMs.

For IFNβ treatment, 70% confluent A549 cells in 24-well plates were treated with IFNβ 500 u/mL (Bio-techne, Abingdon, UK) for 6 h before siRNA transfection with 10 pmol of SMART pool siRNAs specific against human TRIM38 (Catalogue Number: L-006929, Horizon Discovery, Cambridge, UK), human TRIM21 (Catalogue Number: L-006563, Horizon Discovery), human TRIM14 (Catalogue Number: L-010976, Horizon Discovery), or non-targeting (NT) siRNA (Catalogue Number: D-001810-01-20, Horizon Discovery) using 1 µL of lipofectamine RNAiMAX reagent (Invitrogen, Vilnius, Lithuania). After siRNA treatments for 24 h, A549 cells were infected with 0.1 MOI of WNV_KUN_, ZIKV, or LGTV for an additional 48 h, and media from infected cells were harvested.

For immunoblottings, 70% confluent A549 on 12 well plates were transfected with 0.1 or 0.3 ug of TRIM-FLAG constructs or an empty construct as a control, using Lipofectamin 2000 (Invitrogen) with the ratio of the DNA (μg) to the lipofectamine (μL) being 1:2.5. A day after, cells were exposed to LGTV (0.1 MOI) for 48 h prior to assay.

For colocalization studies, transfections of TRIM-FLAG gene constructs were performed according to the manufacturer’s protocol in 8-well Lab Tek II chamber slides (Thermo scientific, Rochester, NY, USA) using Lipofectamine^TM^ 3000 reagent (Invitrogen, Carlsbad, CA, USA) and P3000^TM^ reagent (Invitrogen, Carlsbad, CA, USA) in OptiMEM media for 24 h post-transfection prior to assay. Hereby, 5 × 10^4^ HEK-293T cells on 8 well Lab-Tek II chamber slides were transfected with 250 ng of plasmids DNA for TRIM14-FLAG, TRIM21-FLAG, TRIM38-FLAG, ZIKV NS3-mCh, ZIKV NS5-mCh, LGTV NS3-mCh and LGTV NS5-mCh. Transient overexpressing TRIM-FLAG cells were also exposed to LGTV (5 MOI) for 24 h prior to assay.

The LGTV and WNV_KUN_ replicons were constructed as previously described [32]. The Renilla luciferase-expressing construct (pGL4.74, Promega, Madison, WI, USA) was used for normalized transfections of the LGTV and WNV_KUN_ DNA replicons. 50% confluent A549 cells in 24-well plate were transfected with 50 ng of the DNA replicon plasmids and 15 ng of the plasmid expressing rennila luciferase by Lipofectamin 2000 (Invitrogen) with the ratio of the DNA (μg) to the lipofectamine (μL) being 1:2.5 prior to assay.

### 2.8. Antibodies

The following antibodies were used in this study: mouse monoclonal anti-TBEV E (a gift from United States Army Medical Research, Institute of Infectious Diseases, Fort Detrick, Frederick, MD, USA), mouse monoclonal anti-TBEV NS1 (MyBioSource, San Diego, CA, USA), mouse anti-flavivirus NS1 (Abcam, Cambridge, UK), rabbit anti-WNV NS4A (GeneTex, Hsinchu City, Taiwan), mouse anti-α-tubulin (Invitrogen, USA), and anti-mouse VisUCyte HRP Polymer (R&D Systems, Minneapolis, MN, USA). The following antibody was used for confocal microscopy: anti-chicken custom-generated LGTV NS5 (peptide sequence: CZ-DRHDLHWELKLESSIF) or NS3 (peptide sequence: CZ-RDIREFVSYASGRR) (Aves Labs, Davis, CA, USA), FLAG (Sigma Aldrich, St. Louis, MO, USA) for all FLAG-tagged TRIMs, and dsRed (Takara, San Jose, CA, USA) for mCherry-conjugated ZIKV and LGTV NS3 and NS5, rabbit anti-ZIKV E (GeneTex) and mouse J2 anti-dsRNA (Scicons, Budapest, Hungary) were used as the primary antibodies, whereas Alexa Flour^TM^ 488 goat anti-mouse IgG (H + L) (Invitrogen, Eugene, OR, USA), Alexa Fluor™ 488-conjugated goat anti chicken IgG (H + L) (Invitrogen, Eugene, OR, USA), and Alexa Flour^TM^ 594 goat anti-rabbit IgG (H + L) (Invitrogen, Eugene, OR, USA) were used as the secondary antibodies.

### 2.9. Plaque Assays

Crystal violet-based plaque assay was performed to quantify WNV_KUN_ and ZIKV, whereas immunofocus plaque assay was performed to quantify LGTV. In brief, series of virus dilutions in DMEM were used to infect 90% confluent Vero cells for 1 h at 37 °C, followed by cell-overlaying with DMEM supplemented with 1.2% Avicel (FMC, Philadelphia, PA, USA), 2% HI–FBS (Gibco), 1× non-essential amino acids (Gibco), and 1% PEST (Gibco). After 3–4 days, the overlays were removed, and cells were fixed by methanol for 20 min. The fixed cells were stained by 2% crystal violet (Sigma, Kawasaki, Kanagawa, Japan), 20% methanol (Fisher, Trinidad and Tobago), and 0.1% ammonium oxalate (Sigma) solution. For the immunofocus assay, the fixed cells were blocked by 2% BSA (Fitzgerald Industries International, Acton, MA, USA) for 10 min. Following blocking, the plates were incubated at 37 °C for 1 h with the blocking buffer containing a 1:1000 dilution of mouse anti-LGTV E antibody, followed by a 1:100 dilution of HRP-conjugated anti-mouse secondary antibody. Final staining was performed with KPL TrueBlue Peroxidase Substrate (Seracare, Milford, MA, USA) for 15 min at room temperature (RT).

### 2.10. Immunoblottings

Cells were lysed using 1× LDS sample buffer (Invitrogen). Protein separation was performed on precast 4–12% Bis-Tris polyacrylamide gels (Invitrogen) with MES running buffer (Invitrogen) at a constant voltage of 150 V for 60 min in an ice box. The separated proteins were then transferred onto nitrocellulose membranes using the iBlot 2 Gel Transfer Device (Invitrogen). Target proteins were identified using specific antibodies.

### 2.11. Quantitative Real-Time PCR (qPCR)

Total RNA was isolated from cell lysates and cell culture supernatants using the RNeasy Mini Kit (Qiagen, Hilden, Germany) and QIAamp Viral RNA Mini Kit (Qiagen), respectively. cDNA was synthesized using the High-Capacity cDNA Reverse Transcription Kit (Applied Biosystems, Vilnius, Lithuania). qPCR was conducted using a QuantStudio 7 Flex Real-Time PCR System (Applied Biosystems) with TaqMan Fast Advanced Master Mix (Applied Biosystems) and Custom TaqMan Gene Expression Assays to detect TRIM38, TRIM21, and TRIM14, and GAPDH (Applied Biosystems).

### 2.12. Immunofluorescence Labeling and Confocal Microscopy

Cells grown on coverslips or 8 well Lab-Tek II chamber slides were fixed with 4% paraformaldehyde for 20 min. Cells were incubated with permeabilization buffer (0.1% Triton X-100, 0.1% sodium citrate) for 20 min at RT and incubated with blocking solution (PBS, 0.5% BSA, 1% goat serum) for an additional 30 min. Cells were then incubated with primary antibodies for 1 h at RT, washed three times in PBST (PBS, 0.5% Tween 20), and incubated with secondary antibodies for 1 h. Slides were washed three times in PBST and overlaid with glass coverslips using DAPI Fluoromount-G^®^ media (Southern Biotech, Birmingham, AL, USA). Immunostained cells were visualized using an Olympus Fluoview FV1000 confocal microscope (Tokyo, Japan) or confocal laser scanning microscopy SP8 Leica Microsystems (Wetzlar, Germany). Images were processed and analyzed using ImageJ version 1.54p (National Institutes of Health, Bethesda, MD, USA) and Cellprofiler version 3.0 (Cambridge, MA, USA)

### 2.13. Luciferase Assay

Cell lysates were assayed and measured for bioluminescence using the Dual-Luciferase Reporter Assay Kit (Promega) and a Lumi-star Omega machine (BMG Labtech, Allmendgrün, Ortenberg, Germany), according to the manufacturers’ instructions. Replicon transfection was normalized by co-transfection with a construct expressing Renilla luciferase as an internal control. Replicon expression was quantified as the ratio of firefly luminescence to Renilla luminescence, reported as relative luminescence units (RLUs).

### 2.14. Statistical Analysis

Statistical differences between the means were determined using one-way ANOVA followed by the Bonferroni post hoc test, and *p* < 0.05 was considered a statistically significant difference. GraphPad Prism version 9.0 was used for performing all statistical analyses. The values are presented as mean ± standard error of the mean.

## 3. Results

### 3.1. TRIM Proteins Interact with Flavivirus Replication Complex

**Figure 1:**
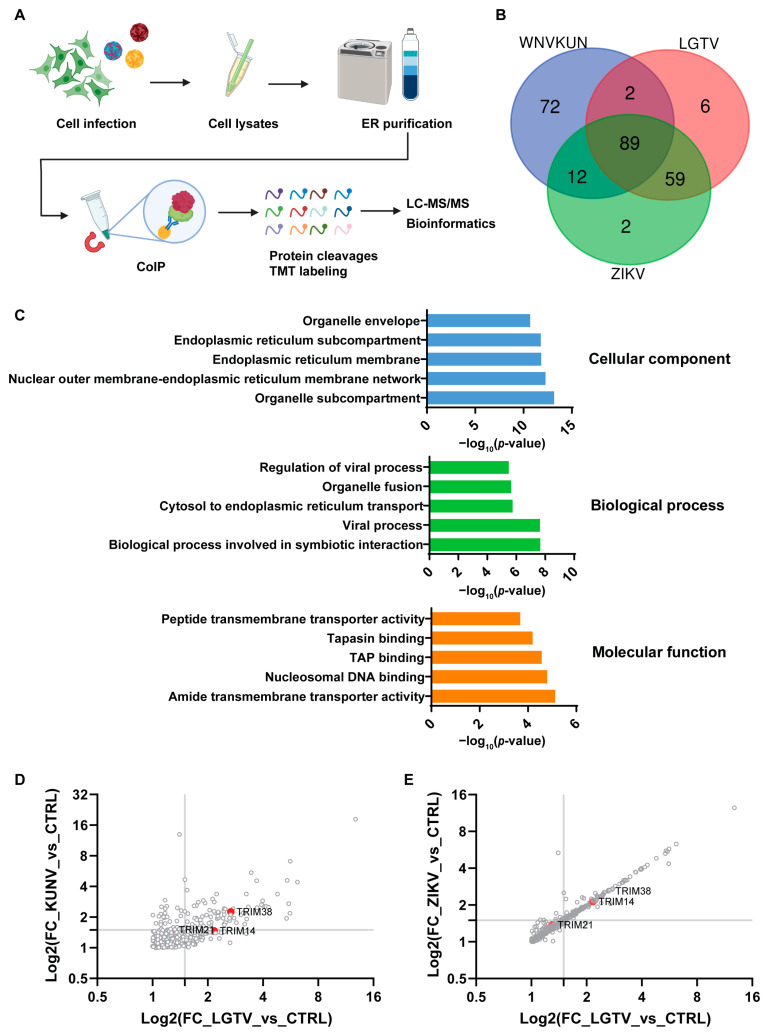
Identification of flavivirus-interacting host proteins, TRIMs. (**A**) Schematic illustration of the experiment procedure to enrich and identify the host proteins interacting with the virus replication complex. The A549 cells were infected with WNV_KUNV_, ZIKV, or LGTV. At 72 h post-infection, cells were mechanically lysed by a Dounce homogenizer. The endoplasmic reticulum (ER) was purified by the ultracentrifugation of the cell lysates on a sucrose gradient, followed by coimmunoprecipitation (IP) with the available NS4A antibodies for WNV_KUN_ and NS1 for ZIKV and LGTV. The pulldown proteins were then cleaved and labeled with tandem mass tags (TMTs) before analysis by liquid chromatography with tandem mass spectrometry (LC-MS/MS). (**B**) Venn diagram showing the unique and overlapping host protein populations from the WNV_KUN_-, ZIKV-, and LGTV-infected A549 cells. Only proteins significantly detected (false discovery rate (FDR): 1%) and fold changes higher than 1.5 in all three replicates in each condition were used in this analysis. (**C**) Gene Ontology (GO) enrichment analysis was performed using ToppFun with the detected proteins (FDR < 0.01 and fold change > 1.5) from the ZIKV-infected ER samples compared to the noninfected ER control. The top five enriched terms are shown in each GO domain. (**D**,**E**) Graph plots of host enriched proteins with the binary logarithm (Log2) of the fold changes (FCs) of the protein abundance in the WNV_KUN_-, ZIKV-, and LGTV-infected samples compared to the noninfected purified ER control. Only proteins with FDRs of 1% are shown. The identified TRIM proteins are highlighted in the graphs.

We infected A549 cells with WNV_KUN_, ZIKV, or LGTV to identify proteins that interact with the viral replication machinery, potentially playing important roles in the infection process. We isolated the ER membrane fraction 72 h post-infection and performed co-immunoprecipitation (IP) using NS4A antibodies for WNV_KUN_ and NS1 antibodies for ZIKV and LGTV. The pulldown proteins were then digested by trypsin and labeled with tandem mass tags (TMTs) before separation and detection by liquid chromatography with tandem mass spectrometry (LC-MS/MS) (Figure 1A). We identified 241 proteins with fold changes higher than 1.5 and false discovery rates (FDRs) less than 0.01 as compared to those at the control ER membrane fraction from the noninfected cells. Out of the 241 proteins, 89 were common in cells infected by each of the three viruses (Table S1). We found a higher number of host proteins that were common between LGTV and ZIKV, compared to WNV_KUN_ and ZIKV, despite WNV_KUN_ and ZIKV being a MBFV and LGTV being a TBFV (Figure 1B). Gene Ontology (GO) enrichment analysis was performed to identify the proteins expressed in the ER membrane during ZIKV infection. As expected, the GO cellular component domain showed that most of the enriched proteins were located at the ER. For the GO biological and molecular process domains, the enriched proteins were involved in regulating viral processes and transmembrane transporter proteins, respectively. Altogether, these data suggest that our experiment procedures strongly enriched the host proteins located at the ER, which may interact with the virus RC (Figure 1C). From the screening, we identified the TRIM38, TRIM21, and TRIM14 proteins, which were enriched during flavivirus infection (Figure 1D,E). We decided to characterize the functions of these TRIMs during flavivirus infection.

### 3.2. TRIM Proteins Are Restriction Factors for ZIKV and LGTV Infection

**Figure 2:**
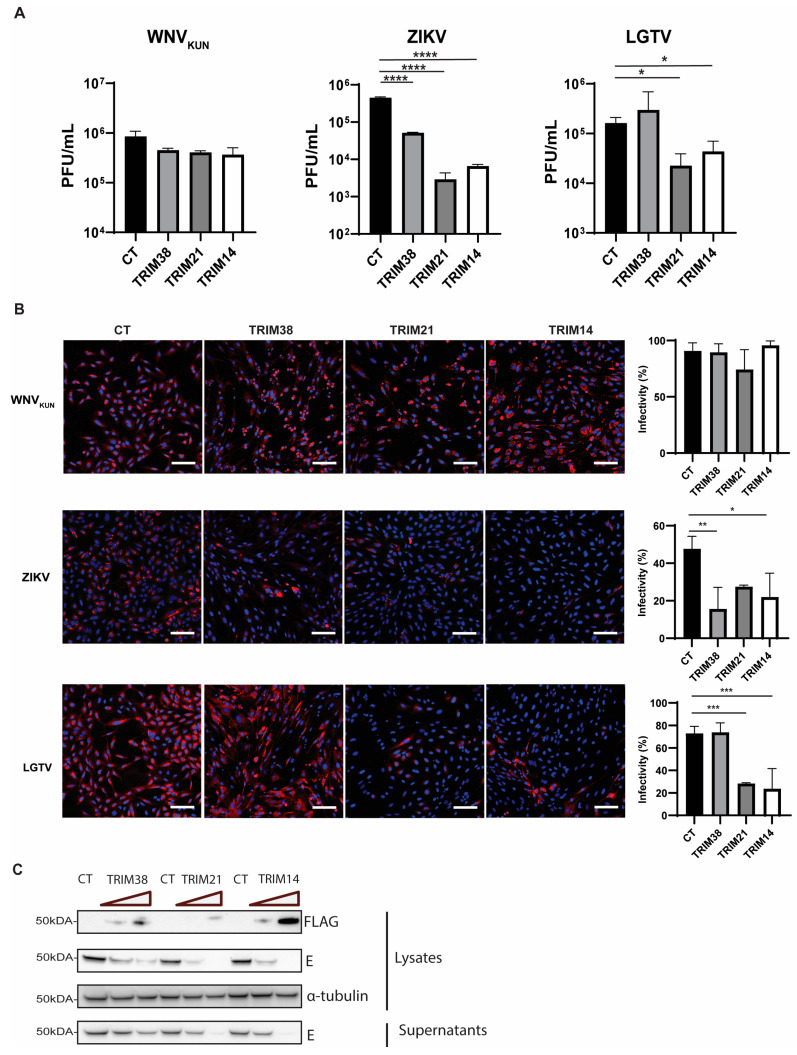
Expression of TRIM38, TRIM21, and TRIM14 restricted ZIKV replication, while LGTV replication was restricted by TRIM21 and TRIM14. (**A**) Virus titers of WNV_KUN_, ZIKV, and LGTV from media 48 h after infecting naïve A549 cells, or cells expressing either TRIM38, TRIM21, or TRIM14, measured by plaque assay. The *p* values are indicated using * *p* < 0.05, ** *p* < 0.01, *** *p* < 0.001, and **** *p* < 0.0001 (**B**) Immunofluorescence microscopy of A549 cells expressing either TRIM38, TRIM21, or TRIM14 infected with WNV_KUN_, ZIKV, LGTV for 48 h with MOI 0.01. The cells were labeled with either the antibody against dsRNA for WNV_KUN_-infected cells or antibodies against the E proteins for ZIKV- or LGTV-infected cells. The nuclei were counterstained with DAPI. Bar scales indicate 100 μm. Cells from the images in the left panel were scored for infection in the right panel using CellProfiler [33]. (**C**) Immunoblotting of cell lysates after transfection with TRIM-FLAG constructs and infection with LGTV. The proteins were visualized with antibodies against FLAG, LGTV E, and α-tubulin as the loading control.

TRIM proteins have been recognized for their antiviral properties for decades. To identify the role of TRIM38, TRIM21, and TRIM14 proteins during flavivirus infection, we made polyclonal A549 cells overexpressing these TRIMs. The cells were infected with WNV_KUN_, ZIKV, or LGTV, and the virus titers in the cell culture media were quantified by plaque assay. Overexpression of these TRIMs demonstrated a significant reduction in the ZIKV and LGTV titers but did not show any significant inhibitory effect on the WNV_KUN_ (Figure 2A). The reduction in the ZIKV titers was 1 log by TRIM38 and 2 logs by TRIM21 and TRIM14. In the case of LGTV, the expression of TRIM38 did not reduce the virus titers, but TRIM21 and TRIM14 reduced the LGTV titers by 1 log (Figure 2A). The effect of these TRIMs on the WNV_KUN_, ZIKV, and LGTV expression was also characterized by staining the virus-infected TRIM-expressing cells with antibodies against dsRNA for WNV_KUN_ and virus E proteins for LGTV- or ZIKV-infected cells (Figure 2B). Similar to the virus titers, the immunofluorescence microscopy showed that the expression of these TRIMs had little impact on WNV_KUN_ infection. Both TRIM21 and TRIM14 restricted the ZIKV and LGTV expression, whereas TRIM38 only inhibited ZIKV. In addition, A549 cells were transfected with escalating concentrations of TRIM-FLAG constructs, followed by infection with LGTV. A marked reduction in the LGTV E protein levels was observed in both the cell lysates and supernatants upon the high expression of TRIM21 and TRIM14, as shown by the immunoblotting (Figure 2C). Thus, these data suggest an antiviral role of the identified TRIM proteins during LGTV and ZIKV infection.

### 3.3. TRIM Proteins Mediate the Antiviral Effects of IFNβ Against LGTV and ZIKV

**Figure 3:**
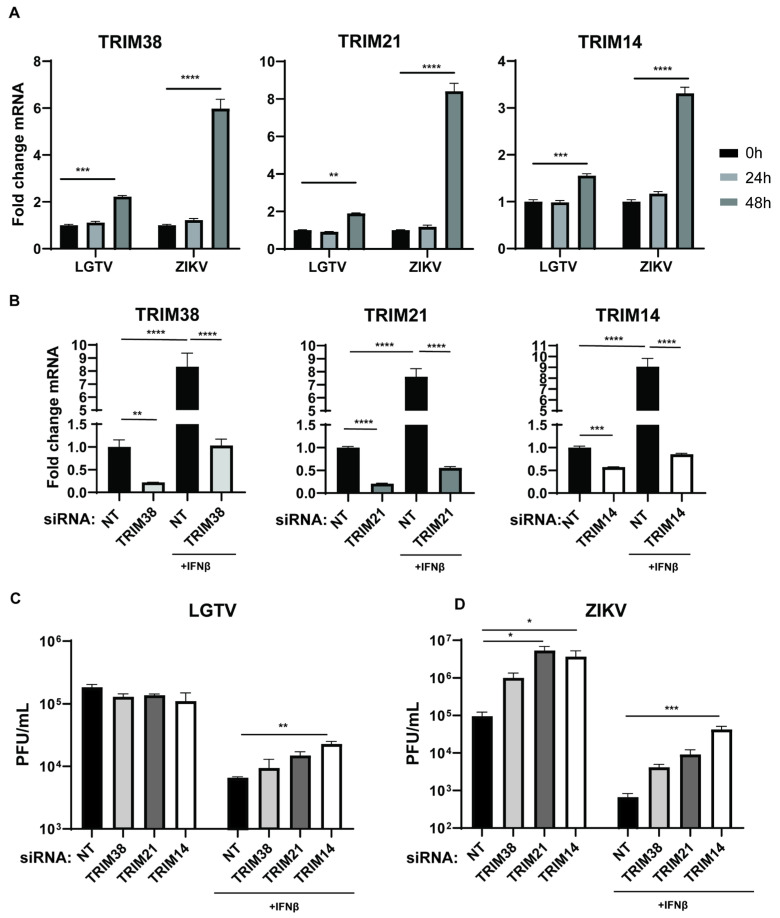
Endogenous TRIM38, TRIM21, and TRIM14 are ISGs with antiviral effects against ZIKV and LGTV. (**A**) Levels of endogenous TRIM38, TRIM21, and TRIM14 mRNA in A549 cells after LGTV or ZIKV infection at 10 MOI. (**B**) Levels of endogenous TRIM38, TRIM21, and TRIM14 mRNA in A549 cells at 72 h post-siRNA transfection. A549 cells were treated with/without interferon (IFN) β for 6 h, followed by transfection with TRIM-specific or non-targeting (NT) siRNA. (**C**,**D**) Virus titers of LGTV and ZIKV in media from A549 cells 48 h post-virus infections and 72 h after transfection with TRIM-specific or NT siRNA. The experiments were conducted independently three times with two technical repeats. The *p* values are indicated using * *p* < 0.05, ** *p* < 0.01, *** *p* < 0.001, and **** *p* < 0.0001.

Many TRIM proteins are expressed as interferon-stimulated genes (ISGs) and play important roles in the host’s immune response to viruses. We characterized the expression of the TRIM38, TRIM21, and TRIM14 genes in LGTV- and ZIKV-infected A549 cells. Infection with both LGTV and ZIKV significantly enhanced the expression of these TRIMs after 48 h (Figure 3A). The increased expression of these proteins could be due to the IFN-I response elicited by the virus infection. In addition, CXCL10 and CXCL11, which are chemokine markers of the IFN-I response, exhibited significantly increased expression during ZIKV and LGTV infection (Figure S1). Notably, ZIKV exhibited a stronger induction of these TRIMs and CXCLs compared to LGTV. To investigate the upregulation of these TRIMs during the IFN-I immune response, we depleted endogenous TRIM proteins by transfecting TRIM-specific siRNA into A549 cells in the presence or absence of IFNβ. The TRIM-specific siRNA treatments resulted in a significant reduction in the mRNA expression for TRIM38, TRIM21, and TRIM14 compared to the NT siRNA. As expected, the expression of these TRIMs in the knockdown cells was enhanced after treatment with IFNβ (Figure 3B). We also infected the TRIM knockdown cells with LGTV and ZIKV, in the presence or absence of IFNβ. In the absence of IFNβ, the TRIM knockdown showed no significant differences in the LGTV titers, whereas the knockdown of TRIM21 and TRIM12 resulted in a significant increase in the ZIKV titers. (Figure 3C,D). These findings suggest that ZIKV could elicit a stronger IFN-I response than LGTV. The depletion of TRIM14 significantly reduced the antiviral effects of IFNβ against LGTV and ZIKV (Figure 3C,D). Altogether, these data suggest that TRIM38, TRIM21, and TRIM14 are ISGs that mediate the IFN-I response against flavivirus infection.

### 3.4. ZIKV NS3 Colocalizes with TRIM38 and TRIM14

**Figure 4:**
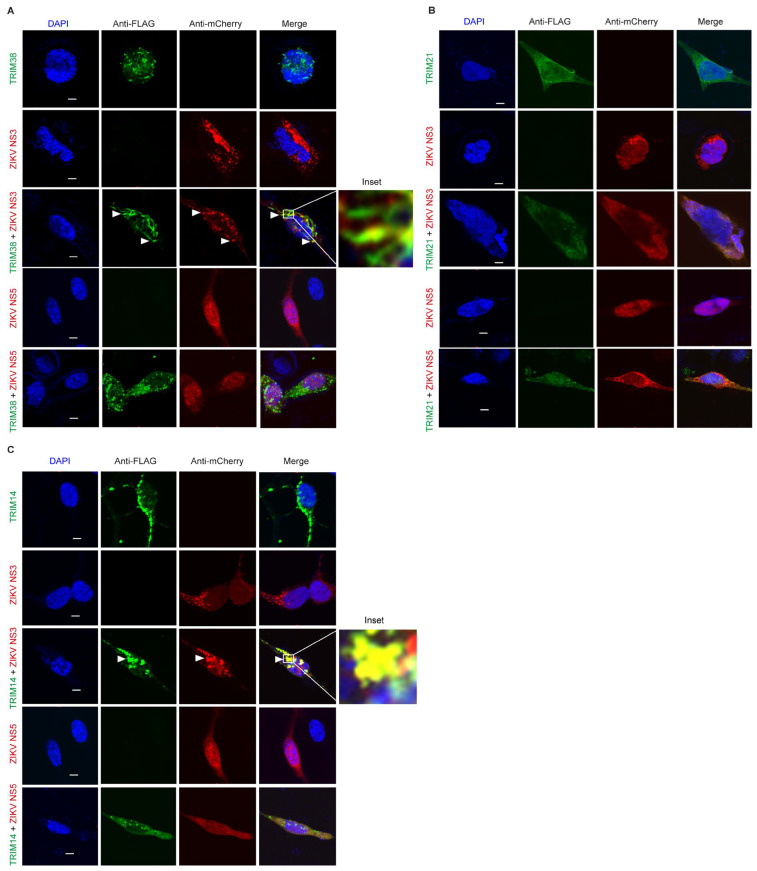
ZIKV NS3 colocalizes with TRIM14 and TRIM38 but not with TRIM21. (**A**) Confocal microscopy of HEK-293T cells expressing TRIM38 with FLAG tag (green) and ZIKV proteins NS3 or NS5 with mCherry tag (red). 5 µm white bars indicate the scale. Arrow heads indicate colocalization site of TRIM38 and NS3. The inset highlights the area of colocalization that is shown at higher magnification. (**B**) Confocal microscopy of HEK-293T cells expressing TRIM21 with FLAG tag (green) and ZIKV NS3 or NS5 with mCherry tag (red). All the experiments were performed in three independent replicates. Nuclei were counterstained with DAPI (blue), and 5 µm white bars are used as the scale. (**C**) Confocal microscopy of HEK-293T cells expressing TRIM14 with FLAG tag (green) and ZIKV proteins NS3 or NS5 with mCherry tag (red). 5 µm white bars indicate the scale. Arrow heads indicate colocalization site of TRIM14 and NS3. The inset highlights the area of colocalization that is shown at higher magnification.

We decided to further explore the antiviral mechanisms of TRIM38, TRIM21, and TRIM14. TRIM proteins often function through protein–protein interactions and largely exert antiviral effects through the targeted degradation of the viral enzymes. The flavivirus NS3 protein encodes a protease and helicase, whereas the NS5 protein encodes methyltransferase and RNA-dependent RNA polymerase, which are essential for virus replication. To investigate how these TRIMs restrict ZIKV and LGTV, we studied the subcellular distribution of these TRIMs and flavivirus NS3 and NS5. In the case of ZIKV, we expressed flag-tagged TRIM38 (Figure 4A), TRIM21 (Figure 4B), and TRIM14 (Figure 4C) alone or in combination with mCherry-tagged ZIKV NS3 or NS5 proteins. When expressed alone, both TRIM38 and TRIM14 were distributed in distinct perinuclear cytoplasmic bodies, whereas ZIKV NS3 displayed a granular distribution. The co-expression of the NS3 protein and TRIM38 or TRIM14 noticeably relocalized the NS3 to large TRIM bodies (Figure 4A,C). The colocalization of ZIKV NS3 with TRIM38 and TRIM14 was specific, as we did not observe any noticeable colocalization of ZIKV NS5 with either of the TRIMs. Interestingly, ZIKV NS5 alone showed a predominant nuclear distribution, but it was translocated from nucleus to cytoplasm in the presence of TRIM21. However, we did not notice any colocalization between TRIM21 and ZIKV NS3 or NS5 under the conditions tested (Figure 4B). Altogether, these results suggest that TRIM14 and TRIM38 restricted ZIKV by relocalizing viral NS3 to the perinuclear TRIM bodies.

### 3.5. LGTV NS3 and NS5 Proteins Are Recruited to TRIM14 Bodies

**Figure 5:**
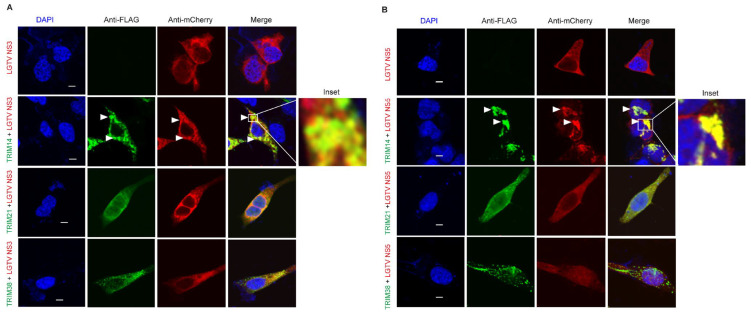
LGTV NS3 and NS5 both colocalize with TRIM14 but not with TRIM21 or TRIM38. (**A**) Confocal microscopy of HEK-293T cells expressing TRIM14, TRIM21, and TRIM38 with FLAG tag (green) and LGTV protein NS3 with mCherry tag (red). Arrow heads indicate colocalization site of TRIM14 and NS3. The inset highlights the area of colocalization that is shown at higher magnification. (**B**) Confocal microscopy of HEK-293T cells expressing TRIM14, TRIM21, and TRIM38 with FLAG tag (green) and LGTV protein NS5 with mCherry tag (red). Arrow heads indicate colocalization site of TRIM14 and NS5. The inset highlights the area of colocalization that is shown at higher magnification. All the experiments were performed in three independent replicates. Nuclei were counterstained with DAPI (blue), and 5 µm white bars indicate the scale.

We next explored the interactions between these TRIMs and LGTV NS3 and NS5. The subcellular localization of flag-tagged TRIM14, TRIM21 and TRIM38 was analyzed in the presence or absence of Cherry-tagged LGTV NS3 (Figure 5A) and NS5 (Figure 5B) proteins. TRIM14 expression colocalized and changed the distribution of both LGTV NS3 and NS5, whereas expression of TRIM38 and TRIM21 did not alter the subcellular distribution of these viral proteins. Since TRIM38 did not restrict LGTV (Figure 2), a lack of colocalization between TRIM38 and LGTV proteins is not surprising. Thus, TRIM14-mediated LGTV restriction coincides with colocalization of both the NS3 and NS5 proteins.

**Figure 6:**
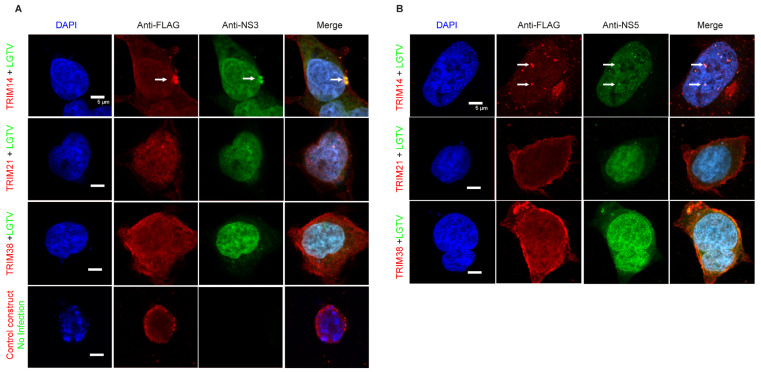
LGTV NS3 and NS5 colocalize with TRIM14 during virus infection. Confocal microscopy of HEK-293T cells expressing FLAG-tag TRIM14, TRIM21, or TRIM38 24 h post-infection with LGTV at a MOI of 5. (**A**) The cells were labeled with anti-FLAG (red), anti-NS3 (green) (**A**), or anti-NS5 (green) (**B**) antibodies. Arrows indicate colocalization site of TRIM14 with LGTV NS3 (**A**) and NS5 (**B**) proteins. Nuclei were counterstained with DAPI (blue), and 5 μm white bars indicate the scale. Images were taken at 100× resolution. All the experiments were performed in three independent replicates.

To determine whether this colocalization is relevant in the context of viral infection, we next examined the colocalization of TRIM with LGTV NS3 and NS5 in virus-infected cells. LGTV infection of TRIM14 expressing cells demonstrated perinuclear and cytoplasmic colocalization between TRIM14 and NS3 (Figure 6A), or within nuclear colocalization with NS5 (Figure 6B). The expression of TRIM21 and TRIM38 did not alter the subcellular distribution of these viral proteins during LGTV infection (Figure 6). Since TRIM38 did not restrict LGTV (Figure 2), the lack of colocalization between TRIM38 and LGTV proteins is not surprising. However, the lack of colocalization between LGTV proteins and TRIM21 (Figure 5 and Figure 6) indicates that the virus restriction (Figure 2) occurs via an alternative mechanism which requires further investigation.

### 3.6. TRIM14 Inhibits LGTV RNA Replication

**Figure 7:**
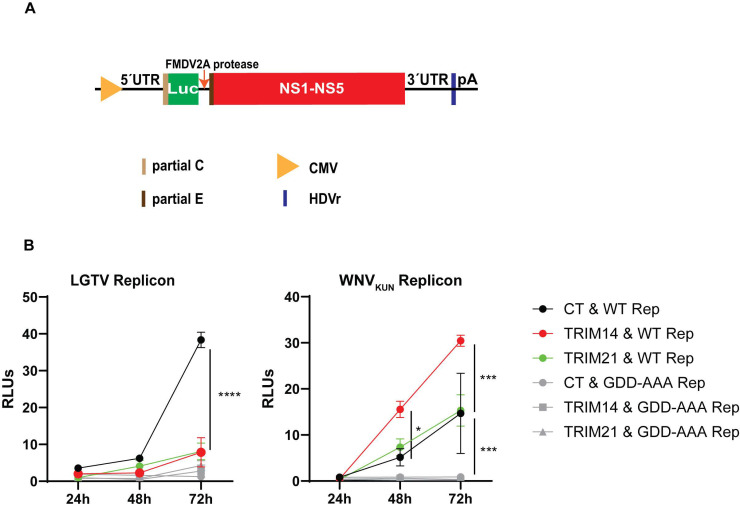
TRIM21 and TRIM14 inhibit LGTV replicon replication. (**A**) Schematic illustration of the flavivirus DNA replicon construct driven by a cytomegalovirus promoter comprising the 5′-untranslated region (UTR), the firefly luciferase gene (Luc) as a reporter gene substituted for most genes coding the structural proteins, the foot-and-mouth disease virus auto-protease 2a (FMDV 2A), all the nonstructural proteins (NS1-NS5), the 3′-UTR, the antigenomic hepatitis delta virus ribozyme (HDVr) sequence, and the simian virus 40 (SV40) polyadenylation signal (pA). (**B**) Relative luminescence units (RLUs) of LGTV and WNV_KUN_ replicons from cell lysates at 24 h, 48 h, and 72 h post-co-transfection of the replicon constructs and the Renilla luciferase construct into A549 cells, or A549 cells overexpressing either TRIM21 or TRIM14. CT: control cells; WT Rep: wild-type replicon; GDD-AAA Rep: replicon with mutations at the GDD motif within NS5. The experiments were conducted independently three times with two technical repeats. The *p* values are indicated using * *p* < 0.05, *** *p* < 0.001 and **** *p* < 0.0001.

Since TRIM14 colocalized with both replication enzymes, NS3 (Figure 5A and Figure 6A) and NS5 (Figure 5B and Figure 6B), we next determined whether TRIM14 targets virus replication. We used our DNA-based LGTV and WNV_KUN_ replicons expressing a firefly luciferase reporter gene (Figure 7A) and replication-deficient GDD replicons as negative controls [32]. Unfortunately, we were unable to generate a functional ZIKV replicon. To determine the effects of TRIM21 and TRIM14 on LGTV replication, A549 cells and TRIM21- or TRIM14-overexpressing A549 cells were transfected with the flavivirus luciferase replicon together with a construct expressing Renilla luciferase as an internal control. The LGTV replication was significantly reduced at 48 h and 72 h post-transfection in cells overexpressing TRIM21 and TRIM14, as compared to normal A549 cells and the LGTV GDD replicon (Figure 7B). In contrast, the WNV_KUN_ replicons showed similar or significantly higher replication in cells overexpressing TRIM21 and TRIM14, respectively, as compared to normal A549 cells (Figure 7B). The reduction in the LGTV replication coincided with TRIM14 colocalization with LGTV NS3 and NS5 proteins (Figure 5), as well as a reduction in the antiviral effects of IFNβ in the absence of TRIM14 (Figure 4C). Altogether, these data suggest that TRIM14 and TRIM 21 restricted the replication of LGTV but not that of WNV_KUN_.

## 4. Discussion

Flaviviruses deform and rearrange the host ER membrane to generate the ER-derived RC, where virus replication and assembly processes take place [4]. In this study, we isolated the ER membrane fraction of flavivirus-infected cells and identified 241 host proteins located at the ER that were enriched during flavivirus infection. The biological process domain of the GO analysis showed that most of the enriched proteins were involved in regulating viral processes, including TRIM38, TRIM21, and TRIM14. In recent decades, viral interactions with human intrinsic and innate defenses have gained increased attention, particularly with the growing interest in TRIM proteins in the context of flavivirus infections. This study characterized the effects of TRIM38, TRIM21, and TRIM14 during WNV_KUN_, ZIKV, and LGTV infections. TRIM proteins are primarily expressed in a constitutive manner and play important roles in intrinsic antiviral immunity, with many TRIMs acting as ISGs [34]. These proteins can not only directly block various stages of virus replication but also stimulate the innate immune response, making them potent broad spectrum antivirals. We observed significant increases in the TRIM38, TRIM21, and TRIM14 expression in A549 cells after ZIKV and LGTV infections, where the former exhibited a stronger effect. In addition, the overexpression of both TRIM21 and TRIM14 led to significant reductions in the ZIKV and LGTV titers, whereas TRIM38 specifically restricted the ZIKV titers. Some viruses have evolved mechanisms to exploit TRIM proteins for their own benefit, suggesting that human TRIM proteins and flaviviruses have coevolved and are probably still engaged in an arms race [35]. This coevolutionary perspective could explain why we did not observe any significant reduction in the WNV_KUN_ titers in cells overexpressing TRIM38, TRIM21, and TRIM14. In addition, the overexpression of TRIM14 led to a significant increase in the WNV_KUN_ replication, and TRIM38 overexpression increased the LGTV titers.

TRIMs are key antiviral restriction factors within the ISG family that play a crucial role in combating viral infection either by directly targeting viral products or by modulating host cellular responses to infection [14,15,16,17,18,19]. Among the 100 TRIMs present in the human genome [21], we previously characterized that TRIM5α and TRIM79α restrict TBFV replication by promoting the proteasomal degradation of viral NS3 and NS5 proteins [22,23]. In the current study, we identified the significant enrichment of TRIM38, TRIM21, and TRIM14 during ZIKV and LGTV infection. We confirmed that these TRIMs are ISGs elicited by the IFN-I response. Notably, ZIKV infection elicited a stronger IFN-I response than that of LGTV infection, as the effect of the TRIM knockdown on the ZIKV titers was visible both in the presence and absence of IFN-β. CXCL10 and CXCL11 are chemokine markers of the IFN-I response, and they are induced by IFN during flavivirus infections [36,37]. Activation of the IFN-I response by LGTV and ZIKV infections was also supported by significant increases in the CXCL10 and CXCL11 expression after virus infection.

Viral enzymes are ideal targets for TRIM-mediated virus restriction, as they are essential for viral replication, protein processing, and assembly. Any disruption of their function can significantly impair the production of new viral particles, making viral enzymes potent antiviral targets [22]. TRIM proteins can recognize specific viral enzymes through protein–protein interactions, enabling targeted degradation that does not interfere with host cell functions [23]. In the current study, TRIM14 colocalized with ZIKV NS3 and LGTV NS3 and NS5. These findings suggest that TRIM14 targets viral enzymes to execute antiviral activity against both ZIKV and LGTV.

Several mechanisms of virus restriction by TRIM14 have been characterized, including the ubiquitination of the IAV nucleocapsid [26], interaction with EBOV NP to enhance IFN-β and NF-κB promotor activation [24], and direct interaction with HBV HBx [38] and HCV NS5A proteins [25]. The expression of TRIM14 significantly reduced the LGTV replication, whereas it enhanced the WNV_KUN_ replication. In addition, the overexpression of TRIM14 led to significant reductions in the LGTV and ZIKV titers. However, TRIM14 colocalized with both the NS3 and NS5 proteins of LGTV, but only with NS3 of ZIKV, thus suggesting a different mechanism for restricting ZIKV. We hypothesize that TRIM14 could target ZIKV NS3 for degradation through polyubiquitination-based pathways. The TRIM14-mediated LGTV replication restriction was linked to its interaction with LGTV NS3 and NS5, indicating these viral enzymes as potential targets of TRIM14. Further studies are needed to explore the degradation of these viral enzymes by TRIM14.

TRIM21 has been shown to restrict virus replication through several mechanisms; for example, it can act as a cytosolic high-affinity antibody Fc receptor to mediate indirect antiviral antibody-dependent neutralization [27]. It facilitates the innate immune response against Newcastle disease virus and Sendai virus via the ubiquitination of mitochondrial adaptor molecules [27]. In addition, TRIM21 initiates a signaling cascade that results in NF-κB activation and the subsequent production of proinflammatory cytokines during virus infection [28], and it inhibits HBV replication by triggering the proteasome degradation of the viral HBx protein [29]. In this study, we found that TRIM21 does not colocalize with NS3 or NS5 of ZIKV or LGTV, despite both viruses being significantly restricted by TRIM21 expression. This lack of colocalization suggests that TRIM21 does not directly bind to NS3 or NS5 but may influence their functional activity through interactions with other viral or host factors essential for viral replication. The role of TRIM21 in RIG-I signaling is well established, and its lack of colocalization with flavivirus NS3 and NS5 may indicate that its antiviral effects occur through the canonical, non-virus-specific pathway [39]. However, our data show that in the presence of TRIM21, ZIKV NS5 translocated from the nucleus to the cytoplasm. This finding suggests that TRIM21 may interfere with the nucleocytoplasmic trafficking of viral proteins, thereby restricting viral replication in a virus-specific manner. The observed specificity of the TRIM21-mediated restriction highlights the ability of the innate IFN response to discriminate between closely related flaviviruses. While TRIM proteins are known to exert antiviral effects through diverse mechanisms, our findings suggest that TRIM21 acts in a virus-dependent manner, potentially through distinct interactions with viral or cellular factors. TRIM38 is one of the three TRIMs that were enriched during flavivirus infection. TRIM38 restricted ZIKV but did not show antiviral activity against WNV_KUN_ or LGTV. The TRIM38 expression was significantly increased after LGTV infection but the overexpression of TRIM38 did not restrict LGTV, and it did not colocalize with either of the viral enzymes.

While it is novel and insightful to identify TRIM21 and TRIM14 as antiviral factors during LGTV and ZIKV infection, we acknowledge certain limitations in our findings. The identification and characterization were conducted using cell lines, which may not entirely reflect the complexity of virus infection dynamics in primary cells and in-vivo. Our colocalization studies provide a limited temporal view, reflecting just one stage of the viral infection process. In this study, we observed that LGTV NS3 localized to the nucleus, consistent with a recent study showing that DENV NS3 can translocate to the nucleus during early infection [40].

Additional studies are required to understand the molecular mechanism behind the TRIM14 and TRIM21 recognition of NS3 and NS5 of ZIKV or LGTV. In addition to their direct roles in virus restriction, TRIM proteins are required to regulate signaling pathways such as RIG-I-like receptors (RLRs) and Toll-like receptors (TLRs) leading to virus detection and innate immune responses [41]. Thus, understanding the precise antiviral mechanisms of TRIM14 or TRIM21 may enable the development of therapeutics effective against ZIKV or LGTV infection. In addition, although hundreds of antiviral genes are expressed in response to IFN, this work demonstrates that antiviral activity can be tailored to individual pathogens by the activity of virus-specific ISGs, such as TRIM proteins. Understanding the complex interactions between TRIMs and viral proteins could lead to the identification of host proteins targeted by flaviviruses for their replication or restriction, which could be exploited for therapeutic strategies.

## Data Availability

The data obtained in this study are available in this article. Supporting data for the published result may be requested from the corresponding author. The database 3112virusSPhuman_20190326 was used. Compiled database from SwissProt: human and 3112_Kunjin virus; 3112_KZVirus_20190322; 3112_Langat virus; 3112_Zika virus strain SL1602.

## Supplementary Materials

The following supporting information can be downloaded at https://www.mdpi.com/article/10.3390/v17050644/s1, Figure S1: Relative expression levels of C-X-C motif chemokine ligand 10 (CXCL10) and CXCL11; Table S1: List of 89 enriched proteins during the virus infection.

## References

[1] ICTV (2021). Genus: Flavivirus. https://ictv.global/report/chapter/flaviviridae/flaviviridae.

[2] Brady O.J., Gething P.W., Bhatt S., Messina J.P., Brownstein J.S., Hoen A.G., Moyes C.L., Farlow A.W., Scott T.W., Hay S.I. (2012). Refining the global spatial limits of dengue virus transmission by evidence-based consensus. PLoS Negl. Trop. Dis..

[3] Apte-Sengupta S., Sirohi D., Kuhn R.J. (2014). Coupling of replication and assembly in flaviviruses. Curr. Opin. Virol..

[4] Kaufusi P.H., Kelley J.F., Yanagihara R., Nerurkar V.R. (2014). Induction of endoplasmic reticulum-derived replication-competent membrane structures by West Nile virus non-structural protein 4B. PLoS ONE.

[5] Miller S., Kastner S., Krijnse-Locker J., Bühler S., Bartenschlager R. (2007). The non-structural protein 4A of dengue virus is an integral membrane protein inducing membrane alterations in a 2K-regulated manner. J. Biol. Chem..

[6] Roosendaal J., Westaway E.G., Khromykh A., Mackenzie J.M. (2006). Regulated cleavages at the West Nile virus NS4A-2K-NS4B junctions play a major role in rearranging cytoplasmic membranes and Golgi trafficking of the NS4A protein. J. Virol..

[7] Lindenbach B.D., Thiel T.H., Rice C.M., Knipe D.M., Howley P.M. (2007). Flaviviridae: The viruses and their replication. Fields Virology.

[8] Zou J., Xie X., Wang Q.Y., Dong H., Lee M.Y., Kang C., Yuan Z., Shi P.Y. (2015). Characterization of dengue virus NS4A and NS4B protein interaction. J. Virol..

[9] Patkar C.G., Kuhn R.J. (2008). Yellow Fever virus NS3 plays an essential role in virus assembly independent of its known enzymatic functions. J. Virol..

[10] Xie X., Zou J., Zhang X., Zhou Y., Routh A.L., Kang C., Popov V.L., Chen X., Wang Q.Y., Dong H. (2019). Dengue NS2A Protein Orchestrates Virus Assembly. Cell Host Microbe.

[11] Zhang X., Xie X., Xia H., Zou J., Huang L., Popov V.L., Chen X., Shi P.Y. (2019). Zika Virus NS2A-Mediated Virion Assembly. mBio.

[12] Wahaab A., Mustafa B.E., Hameed M., Stevenson N.J., Anwar M.N., Liu K., Wei J., Qiu Y., Ma Z. (2021). Potential Role of Flavivirus NS2B-NS3 Proteases in Viral Pathogenesis and Anti-flavivirus Drug Discovery Employing Animal Cells and Models: A Review. Viruses.

[13] Versteeg G.A., García-Sastre A. (2010). Viral tricks to grid-lock the type I interferon system. Curr. Opin. Microbiol..

[14] Best S.M. (2017). The Many Faces of the Flavivirus NS5 Protein in Antagonism of Type I Interferon Signaling. J. Virol..

[15] Chen S., Wu Z., Wang M., Cheng A. (2017). Innate Immune Evasion Mediated by Flaviviridae Non-Structural Proteins. Viruses.

[16] Crook K.R., Miller-Kittrell M., Morrison C.R., Scholle F. (2014). Modulation of innate immune signaling by the secreted form of the West Nile virus NS1 glycoprotein. Virology.

[17] Cumberworth S.L., Clark J.J., Kohl A., Donald C.L. (2017). Inhibition of type I interferon induction and signalling by mosquito-borne flaviviruses. Cell Microbiol..

[18] Schoggins J.W. (2019). Interferon-Stimulated Genes: What Do They All Do?. Annu. Rev. Virol..

[19] Zmurko J., Neyts J., Dallmeier K. (2015). Flaviviral NS4b, chameleon and jack-in-the-box roles in viral replication and pathogenesis, and a molecular target for antiviral intervention. Rev. Med. Virol..

[20] Rajsbaum R., García-Sastre A., Versteeg G.A. (2014). TRIMmunity: The roles of the TRIM E3-ubiquitin ligase family in innate antiviral immunity. J. Mol. Biol..

[21] Han K., Lou D.I., Sawyer S.L. (2011). Identification of a genomic reservoir for new TRIM genes in primate genomes. PLoS Genet..

[22] Chiramel A.I., Meyerson N.R., McNally K.L., Broeckel R.M., Montoya V.R., Méndez-Solís O., Robertson S.J., Sturdevant G.L., Lubick K.J., Nair V. (2019). TRIM5α Restricts Flavivirus Replication by Targeting the Viral Protease for Proteasomal Degradation. Cell Rep..

[23] Taylor R.T., Lubick K.J., Robertson S.J., Broughton J.P., Bloom M.E., Bresnahan W.A., Best S.M. (2011). TRIM79α, an interferon-stimulated gene product, restricts tick-borne encephalitis virus replication by degrading the viral RNA polymerase. Cell Host Microbe.

[24] Kuroda M., Halfmann P.J., Thackray L.B., Diamond M.S., Feldmann H., Marzi A., Kawaoka Y. (2023). An Antiviral Role for TRIM14 in Ebola Virus Infection. J. Infect. Dis..

[25] Wang S., Chen Y., Li C., Wu Y., Guo L., Peng C., Huang Y., Cheng G., Qin F.X. (2016). TRIM14 inhibits hepatitis C virus infection by SPRY domain-dependent targeted degradation of the viral NS5A protein. Sci. Rep..

[26] Wu X., Wang J., Wang S., Wu F., Chen Z., Li C., Cheng G., Qin F.X. (2019). Inhibition of Influenza A Virus Replication by TRIM14 via Its Multifaceted Protein-Protein Interaction With NP. Front. Microbiol..

[27] Li X., Yang L., Chen S., Zheng J., Zhang H., Ren L. (2023). Multiple Roles of TRIM21 in Virus Infection. Int. J. Mol. Sci..

[28] McEwan W.A., Tam J.C., Watkinson R.E., Bidgood S.R., Mallery D.L., James L.C. (2013). Intracellular antibody-bound pathogens stimulate immune signaling via the Fc receptor TRIM21. Nat. Immunol..

[29] Song Y., Li M., Wang Y., Zhang H., Wei L., Xu W. (2021). E3 ubiquitin ligase TRIM21 restricts hepatitis B virus replication by targeting HBx for proteasomal degradation. Antivir. Res..

[30] Luo H., Hu X., Li Y., Lei D., Tan G., Zeng Y., Qin B. (2023). The antiviral activity of tripartite motif protein 38 in hepatitis B virus replication and gene expression and its association with treatment responses during PEG-IFN-α antiviral therapy. Virology.

[31] Williamson C.D., Wong D.S., Bozidis P., Zhang A., Colberg-Poley A.M. (2015). Isolation of Endoplasmic Reticulum, Mitochondria, and Mitochondria-Associated Membrane and Detergent Resistant Membrane Fractions from Transfected Cells and from Human Cytomegalovirus-Infected Primary Fibroblasts. Curr. Protoc. Cell Biol..

[32] Tran P.T., Asghar N., Johansson M., Melik W. (2021). Roles of the Endogenous Lunapark Protein during Flavivirus Replication. Viruses.

[33] Stirling D.R., Swain-Bowden M.J., Lucas A.M., Carpenter A.E., Cimini B.A., Goodman A. (2021). CellProfiler 4: Improvements in speed, utility and usability. BMC Bioinform..

[34] Carthagena L., Bergamaschi A., Luna J.M., David A., Uchil P.D., Margottin-Goguet F., Mothes W., Hazan U., Transy C., Pancino G. (2009). Human TRIM gene expression in response to interferons. PLoS ONE.

[35] Fernandes A.P., OhAinle M., Esteves P.J. (2023). Patterns of Evolution of TRIM Genes Highlight the Evolutionary Plasticity of Antiviral Effectors in Mammals. Genome Biol. Evol..

[36] Indraccolo S., Pfeffer U., Minuzzo S., Esposito G., Roni V., Mandruzzato S., Ferrari N., Anfosso L., Dell’Eva R., Noonan D.M. (2007). Identification of genes selectively regulated by IFNs in endothelial cells. J. Immunol..

[37] Zidovec-Lepej S., Bodulic K., Bogdanic M., Gorenec L., Savic V., Grgic I., Sabadi D., Santini M., Radmanic Matotek L., Kucinar J. (2024). Proinflammatory Chemokine Levels in Cerebrospinal Fluid of Patients with Neuroinvasive Flavivirus Infections. Microorganisms.

[38] Tan G., Xu F., Song H., Yuan Y., Xiao Q., Ma F., Qin F.X., Cheng G. (2018). Identification of TRIM14 as a Type I IFN-Stimulated Gene Controlling Hepatitis B Virus Replication by Targeting HBx. Front. Immunol..

[39] Watkinson R.E., McEwan W.A., Tam J.C., Vaysburd M., James L.C. (2015). TRIM21 Promotes cGAS and RIG-I Sensing of Viral Genomes during Infection by Antibody-Opsonized Virus. PLoS Pathog..

[40] Palacios-Rápalo S.N., De Jesús-González L.A., Reyes-Ruiz J.M., Osuna-Ramos J.F., Farfan-Morales C.N., Gutiérrez-Escolano A.L., Del Ángel R.M. (2021). Nuclear localization of non-structural protein 3 (NS3) during dengue virus infection. Arch. Virol..

[41] Ozato K., Shin D.M., Chang T.H., Morse H.C. (2008). TRIM family proteins and their emerging roles in innate immunity. Nat. Rev. Immunol..

