# Supplementary material for: Identification of TRIM21 and TRIM14 as Antiviral Factors Against Langat and Zika Viruses
Source: Viruses. 2025 Apr 29;17(5):644. doi: 10.3390/v17050644 (PMC12116035; doi:10.3390/v17050644)
Supplement: Supplementary file 1 [file viruses-17-00644-s001.zip › viruses-3513611-supplementary.pdf]

Supplementary Materials

Figure S1. Relative expression level of C-X-C motif chemokine ligand 10 (CXCL10) **(A)** and CXCL11 **(B)** to GAPDH in non-infected cells or cells infected with the viruses. The experiments were conducted independently three times with two technical repeats. The p values are indicated using * $p < 0.05$, ** $p < 0.01$, *** $p < 0.001$, and **** $p < 0.0001$.

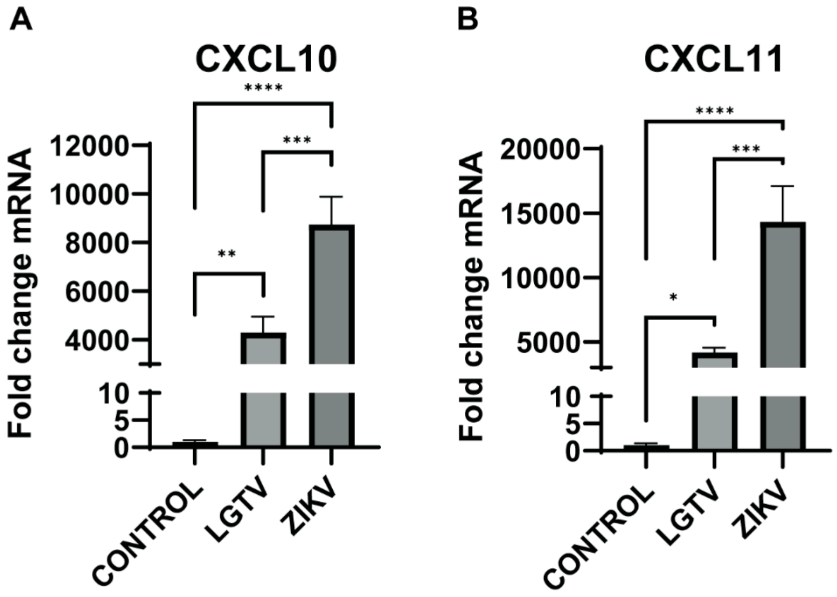

Table S1. List of 89 enriched proteins during the virus infection.

Accession	Description	Gene Symbol
P62306	Small nuclear ribonucleoprotein F	SNRPF
Q96BI3	Gamma-secretase subunit APH-1A	APH1A
Q96LR5	Ubiquitin-conjugating enzyme E2	UBE2E2
P48059	LIM and senescent cell antigen-like-containing domain protein	LIMS1
Q8IZV5	Retinol dehydrogenase 10	RDH10
Q9Y6B6	GTP-binding protein SAR1b	SAR1B
Q7RTP0	Magnesium transporter NIPA1	NIPA1
Q6ZUX7	LHFPL tetraspan subfamily member 2 protein	LHFPL2
Q15800	Methylsterol monooxygenase 1	MSMO1
Q15388	Mitochondrial import receptor subunit TOM20 homolog	TOMM20
P04196	Histidine-rich glycoprotein	HRG
O95299	NADH dehydrogenase [ubiquinone] 1 alpha subcomplex subunit 10, mitochondrial	NDUFA10
P30536	Translocator protein	TSPO
Q08AE8	Protein spire homolog 1	SPIRE1
O15379	Histone deacetylase 3	HDAC3
Q9NVA1	Ubiquinol-cytochrome-c reductase complex assembly factor 1	UQCC; UQCC1
Q9BQN1	Protein FAM83C	FAM83C
Q9C0A0	Contactin-associated protein-like 4	CNTNAP4
O75843	AP-1 complex subunit gamma-like 2	AP1G2
Q13547	Histone deacetylase 1	HDAC1
P55735	Protein SEC13 homolog	SEC13
O15091	Mitochondrial ribonuclease P catalytic subunit	KIAA0391
O14879	Interferon-induced protein with tetratricopeptide repeats 3	IFIT3
P0DJD0	RANBP2-like and GRIP domain-containing protein 1	RGPD1
Q9NWB6	Arginine and glutamate-rich protein 1	ARGLU1
Q96SY0	Integrator complex subunit 14	VWA9; INTS14
P56545	C-terminal-binding protein 2	CTBP2
P57764	Gasdermin-D	GSDMD
Q9HB58	Sp110 nuclear body protein	SP110

Q9BTC8	Metastasis-associated protein MTA3	MTA3
O95674	Phosphatidate cytidylyltransferase 2	CDS2
Q9UL40	Zinc finger protein 346	ZNF346
O00635	E3 ubiquitin-protein ligase TRIM38	TRIM38
Q8IZR5	CKLF-like MARVEL transmembrane domain-containing protein 4	CMTM4
P13645	Keratin, type I cytoskeletal 10	KRT10
Q9BYC8	39S ribosomal protein L32, mitochondrial	MRPL32
Q9P0J0	NADH dehydrogenase [ubiquinone] 1 alpha subcomplex subunit 13	NDUFA13
O00767	Acyl-CoA desaturase	SCD
O75477	Erlin-1	ERLIN1
Q9NZ45	CDGSH iron-sulfur domain-containing protein 1	CISD1
P61619	Protein transport protein Sec61 subunit alpha isoform 1	SEC61A1
P78330	Phosphoserine phosphatase	PSPH
P53701	Cytochrome c-type heme lyase	HCCS
Q7L1V2	Vacuolar fusion protein MON1 homolog B	MON1B
P61204	ADP-ribosylation factor 3	ARF3
O43294	Transforming growth factor beta-1-induced transcript 1 protein	TGFB1I1
O60942	mRNA-capping enzyme	RNGTT
O43169	Cytochrome b5 type B	CYB5B
Q96EI5	Transcription elongation factor A protein-like 4	TCEAL4
P62873	Guanine nucleotide-binding protein G(I)/G(S)/G(T) subunit beta-1	GNB1
Q9NZ42	Gamma-secretase subunit PEN-2	PSENEN
Q92556	Engulfment and cell motility protein 1	ELMO1
P10643	Complement component C7	C7
Q7L775	EPM2A-interacting protein 1	EPM2AIP1
P43307	Translocon-associated protein subunit alpha	SSR1
Q9Y6X4	Soluble lamin-associated protein of 75 kDa	FAM169A
P21926	CD9 antigen	CD9
P16422	Epithelial cell adhesion molecule	EPCAM
Q8TCT8	Signal peptide peptidase-like 2A	SPPL2A
Q14520	Hyaluronan-binding protein 2	HABP2
Q15435	Protein phosphatase 1 regulatory subunit 7	PPP1R7
Q8IWB1	Inositol 1,4,5-trisphosphate receptor-interacting protein 1	ITPRIP
P13929	Beta-enolase	ENO3
P30408	Transmembrane 4 L6 family member 1	TM4SF1
Q14165	Malectin	MLEC
Q6ZPD9	Probable C-mannosyltransferase DPY19L3	DPY19L3
Q9P2X0	Dolichol-phosphate mannosyltransferase subunit 3	DPM3
Q05823	2-5A-dependent ribonuclease	RNASEL
Q9NR31	GTP-binding protein SAR1a	SAR1A
P30479	HLA class I histocompatibility antigen, B-41 alpha chain	HLA-B
Q8WY22	BRI3-binding protein	BRI3BP
P13489	Ribonuclease inhibitor	RNH1
Q96BM9	ADP-ribosylation factor-like protein 8A	ARL8A
P00403	Cytochrome c oxidase subunit 2	COX2
P02794	Ferritin heavy chain	FTH1
Q15629	Translocating chain-associated membrane protein 1	TRAM1
O94808	Glutamine--fructose-6-phosphate aminotransferase [isomerizing] 2	GFPT2
O14524	Nuclear envelope integral membrane protein 1	TMEM194A; NEMP1
Q9UKT5	F-box only protein 4	FBXO4
Q9NYM9	BET1-like protein	BET1L
P04156	Major prion protein	PRNP
Q9NS00	Glycoprotein-N-acetylgalactosamine 3-beta-galactosyltransferase 1	C1GALT1
Q5JSH3	WD repeat-containing protein 44	WDR44
Q96T23	Remodeling and spacing factor 1	RSF1
Q07021	Complement component 1 Q subcomponent-binding protein, mitochondrial	C1QBP
O60237	Protein phosphatase 1 regulatory subunit 12B	PPP1R12B
Q9H853	Putative tubulin-like protein alpha-4B	TUBA4B
Q12840	Kinesin heavy chain isoform 5A	KIF5A
Q66PJ3	ADP-ribosylation factor-like protein 6-interacting protein 4	ARL6IP4
